# Deep learning-based quantitative estimation of lymphedema-induced fibrosis using three-dimensional computed tomography images

**DOI:** 10.1038/s41598-022-19204-6

**Published:** 2022-09-13

**Authors:** Hyewon Son, Suwon Lee, Kwangsoo Kim, Kyo-in Koo, Chang Ho Hwang

**Affiliations:** 1 Major of Biomedical Engineering, Department of Electrical, Electronic and Computer Engineering, University of Ulsan, Nam-gu, Ulsan, 44610 Republic of Korea; 2 Department of Electronic Engineering, Hanbat National University, Yuseong-gu, Daejeon 34158 Republic of Korea; 3 Department of Physical & Rehabilitation Medicine, Chungnam National University Sejong Hospital, College of Medicine, Chungnam National University, 20 Bodeum 7-ro, Sejong, 30099 Republic of Korea

**Keywords:** Anatomy, Medical research

## Abstract

In lymphedema, proinflammatory cytokine-mediated progressive cascades always occur, leading to macroscopic fibrosis. However, no methods are practically available for measuring lymphedema-induced fibrosis before its deterioration. Technically, CT can visualize fibrosis in superficial and deep locations. For standardized measurement, verification of deep learning (DL)-based recognition was performed. A cross-sectional, observational cohort trial was conducted. After narrowing window width of the absorptive values in CT images, SegNet-based semantic segmentation model of every pixel into 5 classes (air, skin, muscle/water, fat, and fibrosis) was trained (65%), validated (15%), and tested (20%). Then, 4 indices were formulated and compared with the standardized circumference difference ratio (SCDR) and bioelectrical impedance (BEI) results. In total, 2138 CT images of 27 chronic unilateral lymphedema patients were analyzed. Regarding fibrosis segmentation, the mean boundary F1 score and accuracy were 0.868 and 0.776, respectively. Among 19 subindices of the 4 indices, 73.7% were correlated with the BEI (partial correlation coefficient: 0.420–0.875), and 13.2% were correlated with the SCDR (0.406–0.460). The mean subindex of Index 2 $\left( {\frac{{P_{Fibrosis\, in\, Affected} - P_{Fibrosis\, in\, Unaffected} }}{{P_{Limb\, in\, Unaffected} }}} \right)$ presented the highest correlation. DL has potential applications in CT image-based lymphedema-induced fibrosis recognition. The subtraction-type formula might be the most promising estimation method.

## Introduction

According to a Lymphoedema Impact and Prevalence International (LIMPRINT) report in 2019, lymphedema can occur in a variety of cancer-associated conditions as well as in heterogeneous medical situations, such as venous diseases and wound-related situations^1^, e.g., cancer manifestation (lymph node metastasis), cancer treatments (radiotherapy and surgery)^2–8^, and soft tissue reconstruction following fracture^9^.

In lymphedema, internally persistent deterioration spontaneously progresses in affected soft tissues, starting first at the molecular level mediated by the increased release of proinflammatory cytokines (interleukin [IL]-6, IL-8, tumor necrosis factor [TNF]-α, transforming growth factor [TGF]-β1) and leading to macroscopic fibrosis consisting of collagen and extracellular matrix deposition^10,11^. It can be visualized using ultrasonography^12,13^ and computed tomography (CT)^14^. Due to the poor capability (29%) of ultrasonography for the visualization of deep tissues in moderate to severe lymphedema (International Society of Lymphology [ISL] stages II and III)^13^, CT can serve as an alternative for visualizing pathological changes within deep tissues^3^. If this internal aggravation can be reliably standardized, patients with lymphedema will be provided with the possibility of early detection and successful management prior to progression into irreversible or nonresponsive stages^6,15^. Fewer patients with disabilities (no less than 75% could maintain their jobs) were observed than expected among lymphedema patients undergoing rehabilitation management, which also supports this demand for early standardized detection regardless of disease duration^1^.

On CT, lymphedema-induced fibrosis is visualized in the form of trabecular reticulation with a honeycomb appearance in subcutaneous fat layers^14,16,17^. Regarding whether CT images could be useful in quantifying histological differences, Geyer et al*.* reported that significant quasilinear correlations were observed with viscoelastic tissue parameters in patients with lipodermatosclerosis^12^. Similarly, significant correlations were observed between the histological degree of fibrosis and CT images in cancer patients with lymphedema and in patients with dermal lymphatic invasion^2,18^. Furthermore, based on the window width of the absorptive values (Hounsfield units [HUs]) in CT scans^19^, fibrosis can be two-dimensionally measured as an area value, with the aid of manual differentiation from other soft tissues and water (for instance, HU of tissue ranges from 20 to 40; HU of water is 0; HU of fat ranges from − 90 to − 70) in cross-sectional images^19,20^. In the late 2010s, the development of a manual tracking-assisted method for the 3-dimensional quantification of fibrosis was reported^21,22^. However, the HU value-based manual tracking method for the quantification of fibrosis showed no correlation with clinical parameters, such as the three-dimensional volume determined by perometry and the ISL stage, and required a considerable amount of time due to the burdensome manual classification process^22^. Therefore, the technically promising results and lack of evidence supporting its applicability in clinical practice necessitate the development of novel image-processing methods.

The concept of machine learning, so called artificial intelligence (AI), was introduced at the Dartmouth Conference in the 1950s. In medical fields, the potential applicability of machine learning was first suggested by Fletcher^23^, and Doi at the University of Chicago widened systematic medical image analysis via machine learning for computer-aided diagnosis and reduction of physician labor in the 1980’s^24^. Since then, machine learning has been extending worldwide into various kinds of medical applications, such as digitized data utilization in healthcare^25^, predictive algorithms in cardiology^26^, and AI-mediated interventions in psychiatry^27^. However, conventional machine learning models have shown insufficient performance and could not satisfy physician demands. To overcome this obstacle, deep learning (DL) has become more common as a solution and involves automatic complex multi-layer neutral network architecture-based learning by converting input data into multiple kinds of abstractions^28^. Regarding DL-based recognition of medical image pattern, convolutional neural network (CNN), which is the most commonly used DL method, can learn automatically how to extract valid features from the training samples for an assigned task by repetitive backpropagation adjustment of its weights without manual designation of features as input information^29^. In terms of high efficacy in the detection and differentiation of specific organs or lesions, DL combined with imaging for diagnosis is of great interest; five-minute magnetic resonance imaging (MRI) sequences augmented by DL superresolution could discriminate synovium, bone, cartilage, and meniscal tissue, with high agreement (54–90% sensitivity and 23–91% specificity) with arthroscopic results^30^. Moreover, combined with positron emission tomography (PET)/CT, DL methods can automatically detect a small nodule within the lungs, even those no less than 2 cm in diameter, with 66.7% sensitivity and 84.5% specificity^31^. Recently, more advanced DL models, called hybrid methods, have been proposed; for example, hybridization of three DL-based algorithms of concatenation, optimization by minimum redundancy maximum relevance (mPMR), and classification by machine learning classifiers showed very high diagnostic yields (accuracy no less than 96.9%)^32,33^.

Based on the finding that HU values of soft tissues are determined by their relative constituents, digital image processing of CT scans can be used to visualize lymphedema-induced fibrosis^21,22,34^. Taking this approach one step further, the authors supposed that the DL-based algorithm could automatically recognize fibrosis from other nonfibrotic soft tissues in cross-sectional CT images. To confirm the validity of the DL-based algorithm in lymphedema patients, the authors compared it with gold standard measurements of lymphedema, such as the results of bioelectrophysiological studies (multiple frequency bioelectrical impedance [BEI] analyses) and the standardized circumference ratio of the affected limbs.

## Results

### Demographic characteristics

**Figure 1:**
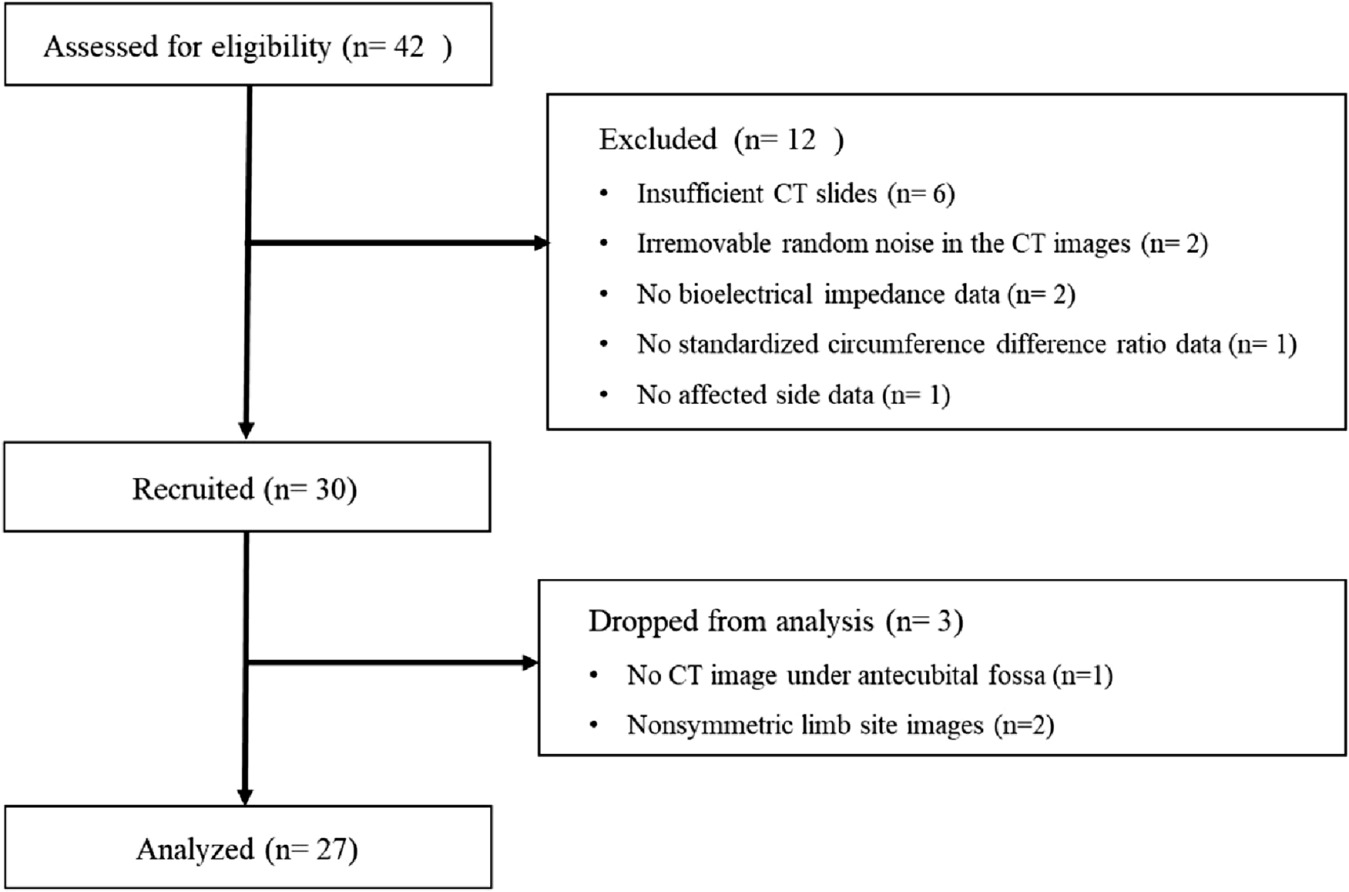
Flow diagram.

Forty-two patients were screened. Among them, 12 patients were excluded due to insufficient CT images (n = 6), irremovable random noise in the CT images (n = 2), no BEI data (n = 2), no SCDR data (n = 1), and no clinical data on the affected side (n = 1). From the 30 recruited patients, 1920 images were acquired and assigned to the training, validation, and test datasets for the proposed model. Additionally, during image calibration for evaluating diagnosis performance of the trained model, data of 3 patients were excluded due to a lack of CT images under the antecubital fossa (n = 1) and nonsymmetrical limb site images (n = 2). Finally, 2138 images of 27 patients were analyzed to compare the proposed indices with the clinical gold standard (Fig. 1). Because some images were excluded for better training but all the images from 27 patients were used to calculate the proposed indices and analyze with the proposed indices, the image numbers for the training are higher even after the three patients were excluded.

The demographic characteristics were as follows. The mean age was 58.38 ± 2.08 years. Most of the patients were female (97.5%); only one patient (2.5%) was male. The mean weight of the patients was 58.67 ± 1.39 kg. Half of the patients (50%) were affected on their left side, and the other half (50%) were affected on their right side. On average, 106.90 ± 18.07 images were collected from each patient. Regarding underlying diseases, postoperative breast cancer was the most common (66.6%), postoperative gynecological cancer was the second most common (29.2%), and primary lymphedema was the third most common (4.2%) disease. CT scanning was performed at a median of 4 years (range, 3–121 months) after the first swelling presentation. None of the patients classified as having ISL stage I or IIIb were included.

### Lymphedema-induced fibrosis labeling

**Figure 2:**
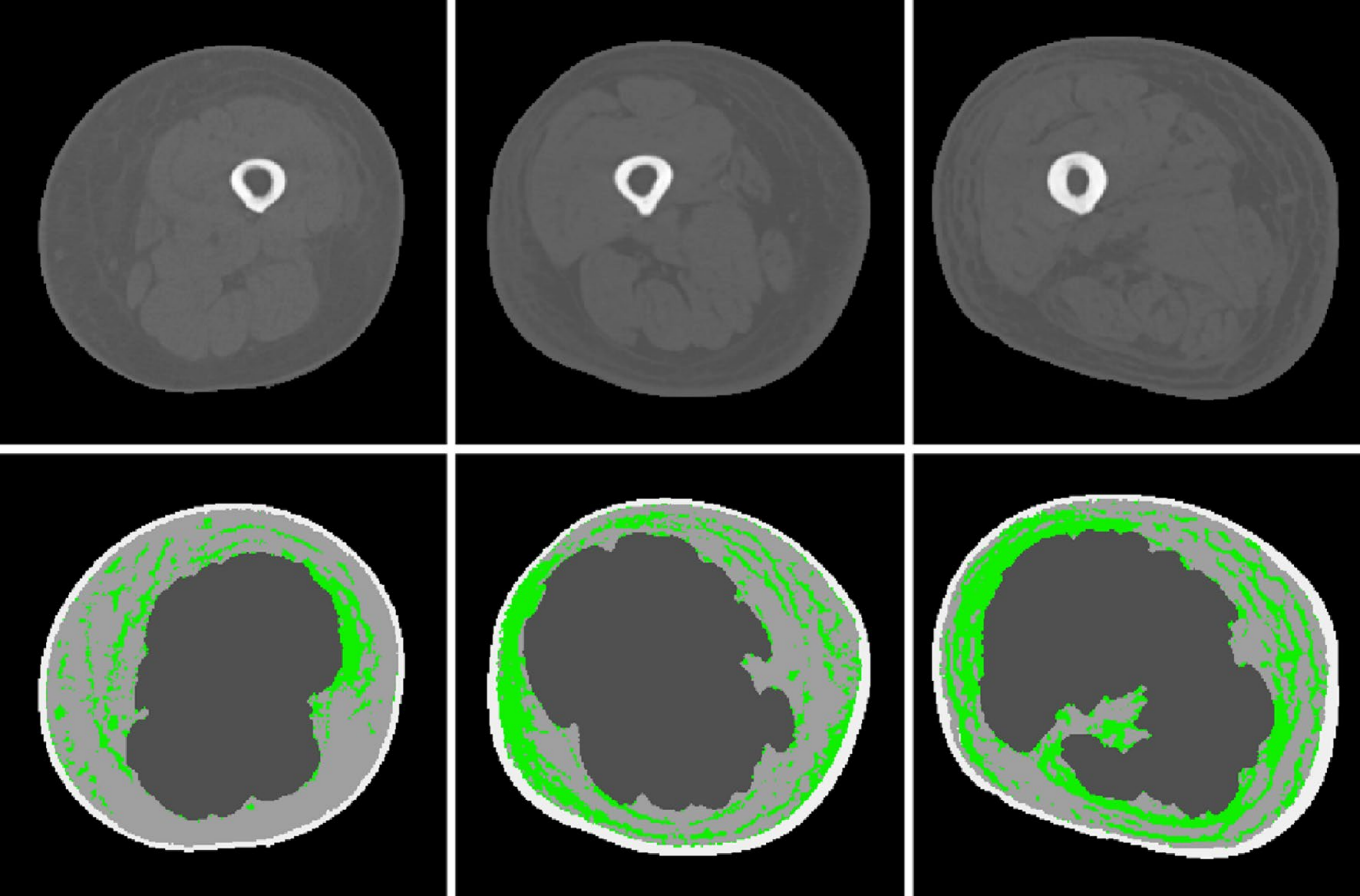
Original CT images (upper row) and images labeled using the lab-developed GUI (lower row). The pixels classified as fibrosis are marked in green.

Figure 2 shows three example images among the original CT images and the images labeled using the lab-developed graphical user interface (GUI).

### DL-based segmentation of lymphedema-induced fibrosis

The segmentation performance of the trained algorithm was evaluated by means of the accuracy, intersection over union (IoU) (also known as the Jaccard index), mean boundary F1 score (MeanBFScore), and Dice similarity coefficient (DSC)^35^. The accuracy is the ratio of the number of correctly classified pixels to all the classified pixels of one label. The IoU is the ratio of the number of correctly classified pixels to the union of pixels with ground truth and all the classified pixels of one label. The MeanBFScore is the average of the boundary scores, *i*.*e*., how well the boundary is classified. Lastly, the Dice similarity coefficient equivalent with the F1 score is calculated by

1 $$ DSC = \frac{2TP}{{2TP + FP + FN}} $$

where TP, FP, and FN represent the number of true positive, false positive, and false negative pixels, respectively. The accuracy, IoU, and DSC for each category were calculated pixel-by-pixel using the confusion matrix in Table 1.

**Table 1:** Confusion matrix of the trained SegNet.

Actual	Predicted				
	Air	Muscle/Water	Fat	Skin	Fibrosis
Air	19,362,257	0	0	9	0
Muscle/Water	601	2,848,499	17,059	977	69,293
Fat	0	33,531	1,190,847	3,354	136,657
Skin	13	6	15,746	447,883	4348
Fibrosis	0	39,396	62,347	42,085	497,700

Air, muscle/water, and skin were fairly well distinguished, with values near 0.9 for all three evaluation parameters, as expected. The fat segmentation accuracy and IoU were less than 0.9. The MeanBFScore of pixels classified as fibrosis was 0.868, but the accuracy, the IoU, and the DSC were 0.776, 0.584, and 0.738, respectively (Table 2).

**Table 2:** Segmentation performance of the trained SegNet.

Class	Accuracy	IoU	MeanBFScore	DSC (F1 score)
Air	0.999	0.999	0.999	0.999
Muscle/Water	0.970	0.947	0.893	0.973
Fat	0.873	0.816	0.921	0.899
Skin	0.957	0.871	0.995	0.931
Fibrosis	0.776	0.584	0.868	0.738

**Figure 3:**
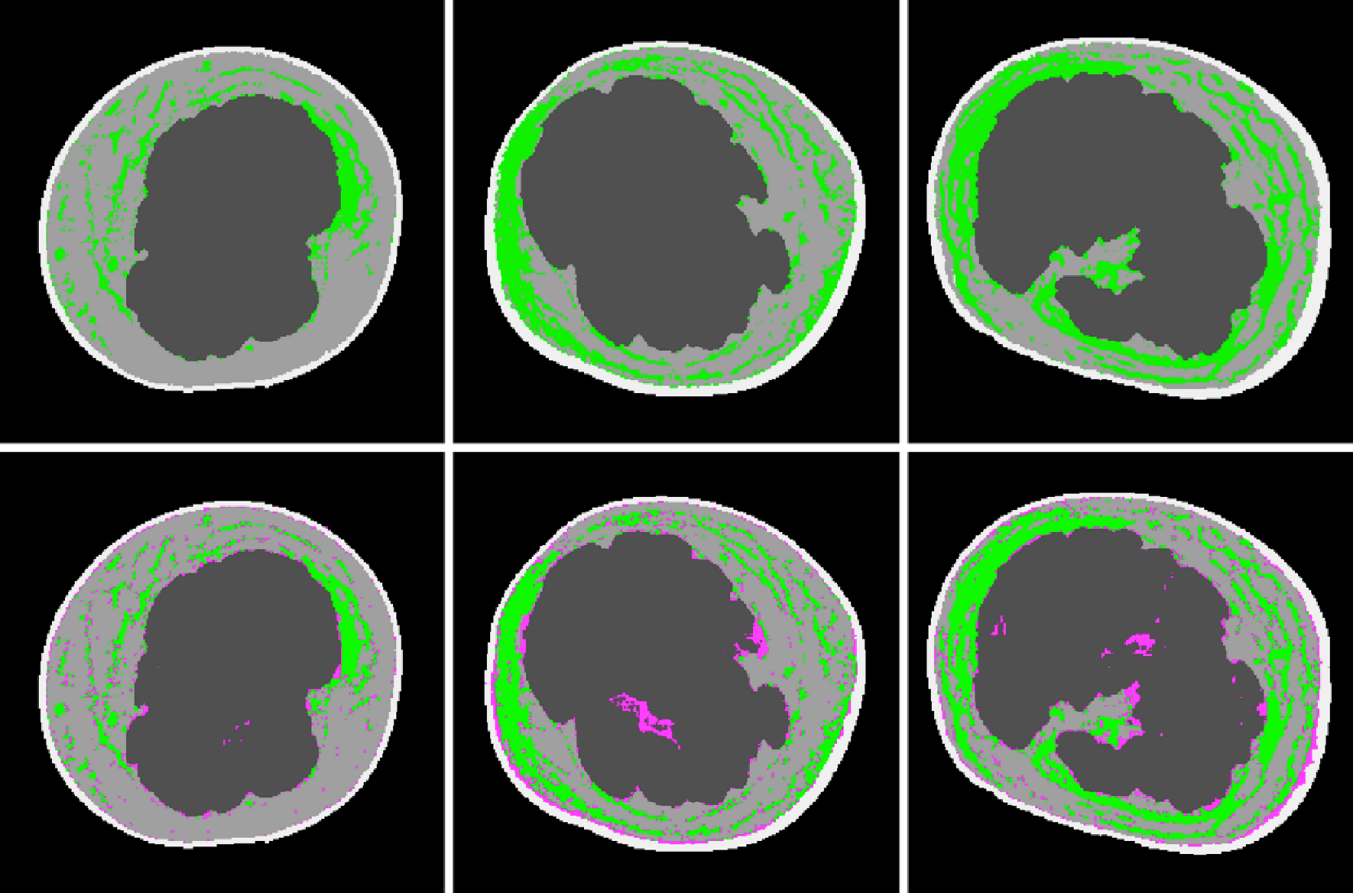
CT images (upper row) labeled using the lab-developed GUI and images classified using the trained algorithm (lower row). The pixels classified as fibrosis are marked in green, and the pixels incorrectly classified as fibrosis are marked in red.

Figure 3 shows three example images among the images labeled using the lab-developed GUI and the images classified using the trained algorithm.

Table 3 shows a comparison of the four indices with two clinical gold standard measurements. Among the 19 subindices, 14 showed a significant correlation with the BEI. The mean subindex of Index 2 in the distal part of the limb (Index 2 _Mean of Distal_), and the BEI showed the highest correlation (0.875). The mean of Index 1 in the distal part of the limb (Index 1 _Mean of Distal_), the mean of Index 2 in the proximal and distal parts of the limb (Index 2 _Mean_), the summation of Index 2 in the proximal and distal parts of the limb (Index 2 _Sum_), the summation of Index 2 in the proximal part of the limb (Index 2 _Sum of Proximal_), the summation of Index 2 in the distal part of the limb (Index 2 _Sum of Distal_), and the mean of Index 2 in the distal part of the limb (Index 2 _Mean of Distal_) showed strong significant correlations with the BEI (partial correlation coefficient > 0.7). However, apart from these three subindices (Index 1 _Mean of Distal_, Index 2 _Mean_, and Index 2 _Mean of Distal_), none showed a significant relation with the SCDR. Overall, the calculated values presented a stronger relationship with the BEI than the SCDR.

**Table 3:** Comparison of the four indices with the clinical gold standard measurements.

Partial correlation coefficient	SCDR_proximal	SCDR_distal	BEI
Index1 _Mean_	0.311	0.274	0.596**
Index1 _Sum_	0.050	0.037	0.508**
Index 1 _Mean of Proximal_	0.020	− 0.027	0.294
Index 1 _Sum of Proximal_	− 0.160	− 0.174	0.326
Index 1 _Mean of Distal_	0.448*	0.427*	0.701***
Index 1 _Sum of Distal_	0.191	0.183	0.552**
Index 2 _Mean_	0.381	0.406*	0.836***
Index 2 _Sum_	0.310	0.304	0.809***
Index 2 _Mean of Proximal_	0.218	0.233	0.630***
Index 2 _Sum of Proximal_	0.281	0.263	0.729***
Index 2 _Mean of Distal_	0.424*	0.460*	0.875***
Index 2 _Sum of Distal_	0.318	0.321	0.832***
Index 3 _Mean_	0.210	0.231	0.223
Index 3 _Sum_	0.176	0.199	0.420*
Index 3 _Mean of Proximal_	0.148	0.175	0.193
Index 3 _Sum of Proximal_	0.060	0.090	0.324
Index 3 _Mean of Distal_	0.239	0.250	0.506**
Index 3 _Sum of Distal_	0.180	0.190	0.546**
Index 4	0.160	0.183	0.551**

*SCDR* standardized circumference difference ratio, *BEI* bioelectrical impedance

*0.01 < *p* < 0.05, **0.001 < *p* < 0.01, ***p < 0.001.

## Discussion

To show the validity of the DL-based algorithm for the automatic quantification of lymphedema-induced fibrosis in CT images, a cross-sectional, observational comparison trial was conducted in chronic lymphedema patients **(**ISL stages II to IIIa**)**. The major findings are as follows: (1) the accuracy and MeanBFScore of the SegNet-based algorithm were 0.776 and 0.868, respectively, showing similar power to previous trials applying a CNN to other diseases; (2) the majority (73.7%) of the 19 subindices of the four indices was significantly correlated with the BEI (partial correlation coefficient: 0.420–0.875), and the minority (13.2%) was significantly related with the SCDR (partial correlation coefficient: 0.406–0.460); and (3) the mean value of Index 2 $\left( {\frac{{P_{Fibrosis\; in\; Affected} - P_{Fibrosis\; in\; Unaffected} }}{{P_{Limb\; in\; Unaffected\;} }}} \right)$ in the distal part of the limb, where the subtraction method was used for standardization, presented the strongest correlation with the BEI.

Ensembles of classifiers demand predefinition, such as feature extraction and region of interest (ROI) definition^36^; thus, low accuracy and incomprehensive results usually occur. Currently, various DL-based algorithms have been developed worldwide because of their strengths (i.e., image segmentation and automated feature generation capabilities)^37^. CNNs, successful DL algorithms based on a multilayer hierarchical network, show high analytical performance when applied to the medical images of patients with various kinds of diseases, such as pulmonary tuberculosis^38^, breast cancer^39^, brain tumors^40^, and hepatic diseases^41^. However, only one study applied a CNN to the quantification of lymphedema-induced fibrosis. Because reliable, repeatable, and highly accurate methods for the early detection of fibrosis (one of the most persistent complications in disease management) would have important clinical significance for the management of lymphedema patients, there is great demand for the establishment of such methods. Positive trials of DL-based image classifiers, such as AlexNet, VGGNet, U-Net, GoogLeNet, SegNet and ImageNet, might lead to fulfillment of this demand. However, in terms of semantic segmentation, U-Net and SegNet are the most popular^42^. SegNet, an automatic image encoder-decoder, was developed for image classification/image segmentation in 2017^43^. It was chosen here because it has simpler layer structure than the U-Net as well as this investigation required pixel-wise semantic classification in the CT image. The authors have plan to implement the proposed segmentation using U-Net and compare each other.

Prior to discussion of SegNet application for CT-based fibrosis correlation with clinical parameters, comparison of the current semiautomatic baseline method on the thing compositions' segmentation might better justify the effectiveness of the proposed workflow. In our previous trial with the same project, in which the current semiautomatic fibrosis segmentation method was used with the same GUI (MATLAB [MathWorks, USA]), 3 types of CT fibrosis index formulated to evaluate their representative capability showed significant correlation with ISL substages (*r* of 0.68–0.79, *p* < 0.01), BEI ratio (*r* of − 0.46, *p* < 0.05), and proximal SCDR (*r* of 0.45, *p* < 0.05) and sensitivity of 0.78 and specificity of 0.60 in lymphatic system dysfunction detection^21^. In terms of CT-based lymphedema fibrosis segmentation, only one report introduced a similar method in which semiautomatic segmentation was conducted by adjusting HU and followed by a convex hull algorithm. Although correlative quantification of fibrosis with a 3-dimensional volume perometry results failed, lateralization of the fibrosis areas was significant in the more-affected limb^22^. Similarly, Edmunds et al*.* conducted CT-based lean muscle area segmentation using semiautomatic calculation of the number of voxels with HU value higher than that of fat. This calculation was followed by smoothing and binning by a non-parametric fitting algorithm for quantification of muscle degeneration. They reported high correlation coefficients (*r* of 0.99, *p* < 0.005) with leg strength, timed up-and go test, gait speed^44^.

In the current trial, in which the image data were divided into training (65%; 1252 images), validation (15%; 290 images), and testing (20%; 378 images) datasets and input into SegNet, the accuracy, IoU, and MeanBFScore for fibrosis were 0.776, 0.584, and 0.868, respectively. Even though the fibrosis accuracy (0.776) is lower than other labels accuracy, it is comparably higher than its random chance (1/5). It was suspected that similarity between the fibrosis and the fat in shape has made it more difficult.

Using the same CNN application, the accuracy of the SegNet-based segmentation model for stroke lesion detection ranged from 85 to 87% in the brain MRI images of stroke patients^45^, in which the dataset contained 420 T1-weighted MRI scans divided into 2 sets for training (70%; 294 images) and testing (30%; 126 images). In another study of 375 patients with 517 focal liver lesions (410 focal liver lesion images for training and 107 focal liver lesion images for testing), the model showed higher accuracy in the detection of hepatocellular carcinoma and benign noninflammatory focal lesions (0.916 and 0.860, respectively) than that in the current trial^46^. However, a different type of CNN, namely, a multiphase convolutional dense network (MP-CDN), was used. In another study using SegNet-based chest X-ray image segmentation (1674 images for training and 199 images for testing), the average accuracy for the detection of a lung nodule and the overlap score were 98.31% and 94.40%, respectively^42^. A comparison trial of a generative adversarial network (GAN) model with a few CNNs (12,150 abdominal CT images for training and 8,800 CT images for testing) showed that the SegNet IoU for hepatocellular carcinoma was 78.57, which is higher than the that in the current trial (0.584)^47^. All of the aforementioned studies, in which stroke lesions, liver masses, or lung nodules were of interest, achieved a higher accuracy and IoU than the current trial, in which fibrotic tissues were of interest. This suggests that the DL-based quantification of lymphedema-induced fibrosis is a greater challenge. Compared to the easy demarcation of oval- or semioval-shaped regions in the abovementioned trials, the difficult demarcation of amorphous fibrotic tissues can explain the lower accuracy and IoU. Moreover, the differences in demographic factors, such as medical diseases, sex, and age, and the relatively small sample size of the current trial should be considered.

Meanwhile, hybrid DL models have shown very successful results in diagnostic accuracy since the early 2020s; in a CNN-based trial of Alzheimer’s disease brain MRI (2560 sections), features were extracted by hybridization of Darknet53, InceptionV3, and Resent101 models and concatenated. Then, they were optimized by mPMR and classified by support vector machine (SVM) and k-nearest neighbors (KNN) and showed very high accuracy of Alzheimer’s disease grades (none, very mild, mild, moderate) ranging from 94.4 to 99.1%^33^. In a CNN-based trial of cystourethrography on pediatric vesicoureteral reflux (1228 images), features were extracted by hybridization of Googlenet, MobilenetV2, and Densenet201 models and concatenated. They then were optimized by mPMR and classified by SVM and KNN and showed very high accuracy of vesicoureteral reflux grades (normal to V) ranging from 95.5 to 96.9%^32^. Regarding the relatively low accuracy in the current trial, the recent hybrid DL models might be a potential solution to increase the accuracy.

**Figure 4:**
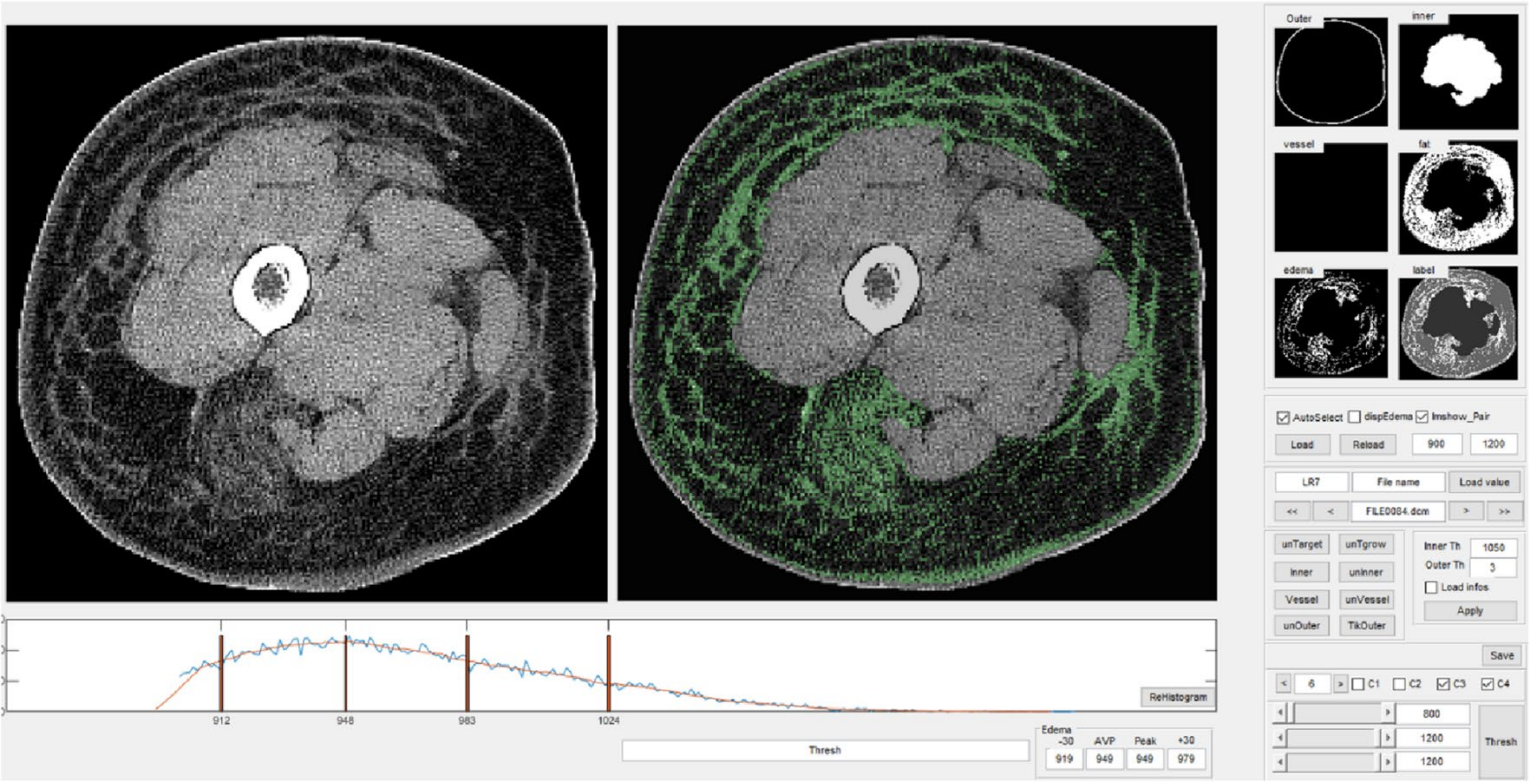
Developed GUI used to categorize every pixel into one of five classes (air, skin, muscle/water, fat, and fibrosis). The pixels classified as fibrosis are marked in green.

In addition to the aforementioned limited accuracy, it is uncertain that the authors measured pure fibrosis. As shown in Figs. 3 and 4, what the authors called "fibrosis" is areas with non-fat attenuation in subcutaneous fat layers. Such areas might include fibrosis but also can include moderately sized vessels and lymphedema itself. The authors are aware of the inclusion of vessels and acknowledge that blood and surrounding vessels in the donut-shaped region were labeled fibrosis based on the assumption that the total number of blood vessel pixels (the total area occupied by blood vessels) was nearly the same in the affected and unaffected limbs.

The most significant problem is that the authors cannot discriminate between lymphedema and fibrosis. Fibrosis involves macroscopic change that can be visualized on histo-pathology^10^. As shown in previous reports^14,16,17^, fibrosis can be viewed on conventional-resolution CT at a 512 × 512 pixel matrix size and an element size of 0.5 mm (0.25 mm in super-high resolution)^48^. Thus, fibrosis is measurable by current DL-based segmentation. Meanwhile, exudate fluid in pure lymphedema without fibrosis can induce reticulation in CT scanning^49^. So the reticulation in the subcutaneous fat compartment usually contains both water and fibrosis. HU value for suprafascial lymphedema is known to range from − 120 HU to − 100 HU^50^. However, the HU was measured a few times at selective areas within subcutaneous fat compartments with no discrimination of lymphedema from the fat in such reports. Furthermore, fat infiltration, an inevitable complication in lymphedema^10^, cannot be helped but included as well in such assessments. As the results, both background fat and fat infiltration cause shifting of the selected ROI value down toward that of fat (− 70 HU to − 190 HU)^34^, similar to Edmunds et al.’s report^44^ and Aubrey et al.’s report^51^. However, if this affection is abolished with the aid of the method in the current study, value of the selected ROI will return to the original value of soft tissue (30 HU to 60 HU)^34,51^. In addition, muscles in general show CT value of 30 HU to 60 HU in healthy young population^51^. However, low attenuation of muscles (− 29 HU to 29 HU) can happen in case of muscle-affecting focal/systemic diseases: for instance, denervated muscles and muscles in the elderly. In such cases, fat infiltration is the most acceptable factor as an underlying cause^51^. Similar to that, it is well known that fat infiltration occurs inside the affected muscles as the results of lymphedema-induced inflammatory cascade. Considering the aforementioned two findings, authors selected pixels of HU from − 34 to 26 to remove the fat-infiltrated, low attenuated muscles and interstitial water (HU: 0) inside the lymphedema, and then reticulation pixels were differentiated, via k-means clustering method, into either of the subcutaneous fat and the fibrosis in the current study.

However, lymphedema is defined as accumulation of tissue fluid in the extracellular, interstitial space and can be observed only at a microscopic level. CT, including super-high-resolution CT, cannot yet visualize a microscopic scale. Following the pattern of improved MRI resolution to 7.0 T and 11.74 T^52,53^, the authors expect advances in CT technology to allow visualization of lymphedema.

In contrast to fibrosis segmentation performance, the correlation of the calculated indices with the clinical gold standard measurements showed more advanced outcomes (Table 3); previous CT image values showed a correlation coefficient of 0.46 with the 1-kHz-based BEI ratio and of 0.45 with the SCDR in a manually predefined segmentation study of lymphedema-induced fibrosis^21^. The 0.45 correlation coefficient with the SCDR is similar to that in the current trial (0.448 for the proximal SCDR and 0.406 for the distal SCDR). However, the correlation coefficient (0.875) with the 1-kHz-based BEI ratio in the current trial is much higher than that (0.46) in the former trial. Considering that the purpose of the current trial is to develop a DL-based algorithm as an alternative to traditional filter-based algorithms demanding manual predefinition and feature extraction, this advancement suggests the superiority of CNN over traditional methods in automatic lymphedema-induced fibrosis recognition.

In lymphedema, inflammatory fibrosis is first triggered at the cellular-molecular level via increased transcription of IL-6, IL-8, TNF-α, and TGF-β1. If lymphatic system dysfunction does not spontaneously recover or is well compensated following this trigger in the acute period^54–56^, lymphoproliferative inflammation will continue to worsen over time. This will result in macroscopic fibrosis consisting of collagen and extracellular matrix deposition^10,11^. Lymphedema-induced fibrosis takes place internally between the skin and muscle layers through replacement of normal anatomical components by fibrotic tissues rather than outward expansion of the limb volume^10,11^. Moreover, this reconstitution change occurs dominantly in subcutaneous fat layers^57^ in the form of a spatiotemporal cascade^22^. Therefore, a simple measurement of the external circumference cannot precisely represent this kind of inner fibrotic replacement. Furthermore, adiposity can be affected by anatomical location and sex^58^; the larger is the area of the involved subcutaneous fat compartment in an individual patient, the greater can be the discrepancy between the inner change and external limb circumference. Taking the above findings into consideration, a lower correlation of the four indices with the SCDR might be expected. Moreover, the low-frequency direct current used in BEI analysis is mainly conducted via interstitial fluid^59,60^; in other words, the impedance value measured by BEI analysis, which is dependent on the relative ratio of connective tissue, in which electrical current cannot propagate as efficiently as in the interstitial space, could be more suitable for representing inner fibrotic changes than the external circumference^61^. Considering the above findings, the high correlation of the four indices with the BEI might be expected. However, further comparison studies involving direct histological findings, such as those of tissue biopsies, might be required for more definite validation.

All the subindices of Index 2 showed moderate to strong significant correlations with the BEI (0.630–0.875). However, only half of the Index 1, 3, and 4 subindices showed moderate significant correlations (0.420–0.701). This discrepancy was remarkable. Because the pixels classified as fibrosis in the affected limb were subtracted from the pixels classified as fibrosis in the unaffected limb, some segmentation errors in the affected limb might have been eliminated, potentially contributing to the stronger correlation. In image-based medical trials, due to common phenomena of anatomical variation among individuals^62^, use of the relative ratio rather than the absolute ratio is the preferred data standardization technique. Therefore, Index 2 calculated using the above standardization method might demonstrate higher classification power than Indices 1, 3, and 4, such that it would show the highest relationship with the BEI, similar to a calculation formula used in another image segmentation-based AI lymphedema study^21^.

### Limitations

Instead of an intention-to-treat analysis, a per-protocol analysis was used in the current trial because the protocol was violated in 3 patients. The effect size can be overvalued in per-protocol analyses^63^. Moreover, if the sample size is small, this phenomenon can be stronger. As a widely reported DL-related issue, overfitting (good fit on the training dataset but poor performance on a new test dataset) should be considered^64^. Because images from 30 patients (1920 CT images) were utilized for the model development in the current trial, actions to avoid overfitting, such as normalized layer generation, dropout layer insertion, and data augmentation to compensate for data variations^46^, had to be conducted. Because images can be influenced by CT scanning parameters or acquisition protocols at individual medical centers, using the current model with a single CT scanner at a single center could lead to data and/or model bias. Another limitation is that female patients made up the vast majority of the study population (only one patient was male)^65^. Additionally, the study followed a cross-sectional rather than prospective design. Although lymphedema-induced subfascial muscular fibrosis^66^ and subsequent muscular change^44^ have been reported, they were not included here. Future studies are needed to support the current findings in a larger number of subjects.

## Conclusions

To show the validity of the DL-based algorithm for identifying lymphedema-induced fibrosis in CT scans, a cross-sectional, observational cohort trial was conducted, and the proposed algorithm was compared with the two gold standard measurements of lymphedema: the BEI and SCDR. In total, 1920 images of 30 lymphedema patients were assigned to a training dataset (65%), validation dataset (15%), and test dataset (20%). The accuracy and MeanBFScore of the SegNet-based fibrosis segmentation were 0.776 and 0.868, respectively. The 4 calculated indices showed a significant strength of correlation (0.420–0.875) with the BEI in patients with chronic moderate-to-severe lymphedema. Among the 4 calculated formulae, the subtraction-type formula is the most promising for this estimation and could act as a foundation for development of automatic recognition systems for fibrotic changes resulting from various inflammation-provoking diseases, such as cellulitis and generalized edema^67^. Such systems would use cross-sectional CT images, leading to a greater chance of early detection with standardized classification in the future.

## Methods

A cross-sectional, observational cohort trial was conducted from January 2018 to March 2019 at a teaching university hospital/tertiary medical center. The protocol of this study was approved by the University Hospital Institutional Review Board (2018-04-009) and was registered at the Protocol Registration and Results System (PRS), www.clinicaltrials.gov (NCT04811677: https://clinicaltrials.gov/ct2/show/NCT04811677?term=NCT04811677&draw=2&rank=1). All methods were performed in accordance with the relevant guidelines and regulations. The trial conformed to the tenets of the Declaration of Helsinki. Patients were included if they were clinically diagnosed with unilateral limb lymphedema and had undergone BEI analysis and CT scanning. The subjects provided written informed consent for publication of the case details. Data were collected as close to the CT scanning date as possible. Patients who were diagnosed with deep vein thrombosis, bilateral limb involvement, vascular disease, or local infection were excluded.

### Bioelectrical impedance (BEI) analysis at 1 kHz

Multifrequency BEI analysis and BEI spectroscopy are frequently used to verify the interstitial fluid of patients with lymphedema^59,60^. Regarding accuracy, the lower is the frequency, the more reliable is the measurement^59^. However, a frequency of 0 kHz is not applicable, so BEI analysis was conducted at the lowest appropriate frequency (1 kHz)^60^. To minimize variation in the interstitial hydrostatic pressure^68^, patients were requested to maintain their regular diet the day before the BEI analysis and to rest in a supine position without movement for 20 min before the analysis. The data were collected using the direct segmental measurement BEI analysis method. The value on the affected side was subtracted from the value on the unaffected side, and that result was divided by the value on the unaffected side.

### Standardized circumference difference ratio (SCDR)

After the patients were instructed to lie in the supine position, the limb circumference 5 cm below and 5 cm above the midpopliteal fossa or midantecubital crease was measured on both sides with nonelastic 1-mm polyvinyl chloride fiberglass tape. The difference between the affected and unaffected sides was divided by the circumference of the unaffected side.

### Lymphedema-induced fibrosis labeling

In this trial, an automatic segmentation method using the DL algorithm was applied. The patients were instructed to keep their body in an anatomically neutral position with both arms in full extension if necessary. Both limbs from the wrist crease to the distal tip of the clavicle or from 5 cm above the lateral malleolus to the point of lower limb separation were simultaneously scanned using CT^21,69^. Among them, images of the patellae were manually excluded because their composition prohibits fibrosis. The CT images were acquired by a single 64-channel, multi-detector plain CT (GE Discovery CT 750 HD, GE Healthcare, USA), parameters of which are 120 kVp, pitch of 0.984 : 1, 64 × 0.625 collimator configuration, slice thicknesses of 3.8–5.7 mm, and 120 mA. The data were preprocessed at a 512 × 512 pixel matrix size and element size of no larger than 0.5 mm.

**Figure 5:**
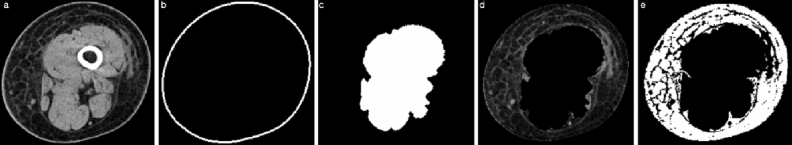
Labeling process using the lab-developed GUI. (**a**) Original image. (**b**) Selection of exterior skin pixels. (**c**) Manual selection of interior muscle and water pixels from − 34 to 26 HU. (**d**) Selection of the donut-shaped area in the original image using the labeled skin and muscle/water pixels. (**e**) Differentiation of fat and fibrosis pixels using the k-mean clustering method.

The collected CT images were labeled semiautomatically using a lab-developed MATLAB (MathWorks, USA) GUI, as shown in Fig. 4. Figure 5 shows step-by-step images of the semiautomatic labeling process. The original image (Fig. 5a) was binarized, and then the inside of the largest region was eroded to label the skin pixels (Fig. 5b). Pixels from − 34 HU to 26 HU in the original image were selected manually to identify the muscle and water pixels (Fig. 5c). In the original image, the labeled skin and muscle/water pixels were subtracted from the remaining donut-shaped area (Fig. 5d) where the fat and fibrosis pixels were combined. The pixels in the donut-shaped region were then identified as fat or fibrosis pixels using the k-means clustering method, an unsupervised learning method. As a result, every pixel in the original CT images was labeled air, muscle/water, skin, fat, or fibrosis for training. Blood and surrounding vessels in the donut-shaped region were labeled fibrosis instead of being distinguished as blood vessels based on the assumption that the total number of blood vessel pixels (the total area occupied by blood vessels) was nearly the same in the affected and unaffected limbs.

### Algorithm training

As a DL model for pixelwise segmentation, SegNet was chosen and trained using 1920 CT images. It is one of the most common models used for semantic segmentation in digital images. As the input, the original gray-scale CT images were cropped to 256 by 256 pixels. Among the 1920 cropped images, the proportions of images selected for the training dataset, validation dataset, and test dataset were 65% (1252), 15% (290), and 20% (378), respectively. All the images from one patient were assigned to the same dataset. By this method, images from one patient were not used for training and testing simultaneously. The training and validation datasets were augmented with translation, reflection, and rotation. Every image in the training dataset was translated from − 3 to 3 pixels along the X or Y direction, reflected to the horizontal direction, and rotated between − 30 degrees and 30 degrees at an interval of one degree. By this augmentation approach, the collective training and validation datasets were increased to 4,684,596 images. Using the augmented images, the SegNet-based model was trained with an encoder depth of 3, 55 epochs, learning rate of 0.002, a minibatch size of 16, the Adam optimizer, and shuffling after every epoch. The encoder depth of 3 indicated 3 sets of one convolution layer and one ReLu and one max pooling layer with 2 strides for down sampling. After encoding, 3 sets of up sampling layers were arranged. A commercial mathematical package, MATLAB (MathWorks, USA), in a graphics processing unit environment (GeForce RTX 2070 SUPER, NVIDIA, USA) was used to train the SegNet-based model.

### Data analysis

Based on the segmentation results of the trained algorithm, four types of indices were defined and calculated for every cross-sectional CT image for comparison with the clinical gold standards, i.e., the BEI and SCDR. The first index was defined as the ratio between the affected and unaffected limbs of the summation of the fat- and fibrosis-classified pixel numbers, as shown in Eq. 2.

2 $$ \frac{{P_{Fat\; in\; Affected} + P_{Fibrosis\; in\; Affected} }}{{P_{Fat\; in\; Unaffected} + P_{Fibrosis\; in\; Unaffected} }} $$

where $P_{Fat\; in\; Affected}$ is the number of pixels classified as fat in the affected limb, $P_{Fibrosis\; in\; Affected}$ is the number of pixels classified as fibrosis in the affected limb, $P_{Fat\; in\; Unaffected}$ is the number of pixels classified as fat in the unaffected limb, and $P_{Fibrosis\; in\; Unaffected}$ is the number of pixels classified as fibrosis in the unaffected limb. Technically speaking, a pixel classified as fibrosis should be reticulated only in the affected limb. Although reticulations with a honeycomb appearance are the result of lymphedema-induced microscopic inflammation in the subcutaneous layers, as described in the Introduction^14,16,17^, pixels classified as fibrosis were present in the unaffected limb and might be representative of subcutaneous fat septa or superficial fascia^67^. However, for notation uniformity, these pixels are denoted as $P_{Fibrosis\; in\; Unaffected}$.

The second index was defined as the difference between the number of pixels classified as fibrosis between the affected and unaffected limbs divided by the number of pixels in the unaffected limb, as shown in Eq. (3).

3 $$ \frac{{P_{Fibrosis\; in\; Affected} - P_{Fibrosis\; in\; Unaffected} }}{{P_{Limb\; in\; Unaffected\;} }} $$

where $P_{Limb\; in\; Unaffected}$ is the total number of pixels classified as skin, muscle/water, fat, or fibrosis in the unaffected limb.

The third index was defined as the ratio of the number of pixels classified as fibrosis in the affected and unaffected limbs, as shown in Eq. (4).

4 $$ \frac{{P_{Fibrosis\; in\; Affected} }}{{P_{Fibrosis\; in\; Unaffected} }} $$

The fourth index was defined as the sum of all the pixels classified as fibrosis in the affected limb of one patient, as shown in Eq. (5).

5 $$ \Sigma P_{Fibrosis\; in\; Affected} $$

### Statistical analysis

The sample size was calculated using Power Analysis and Sample Size Software, version 11.0. (PASS, NCSS Statistical Software, Kaysville, UT, USA: https://www.ncss.com/software/pass/). For two correlation analyses, a power of 0.80, an α of 0.05, an R0 (baseline correlation) of 0, and an R1 (alternative correlation) of 0.5 were chosen based on data reported by Koo et al*.*^21^. The estimated minimum sample size was 29^70^. Considering that image processing-based trials are well controlled, the likelihood of midterm dropout was expected to be very low. Allowing for a dropout ratio of 5%, 30 patients were required. Two-sided statistical analyses were conducted using the Statistical Package for the Social Sciences, version 24 (SPSS, Inc., Chicago, USA). Normality verification was performed using the Kolmogorov–Smirnov test. To remove the linear effect derived from interstitial fluid (BEI) or limb swelling (SCDR), the four indices were compared with the BEI and SCDR using partial correlation analysis. If a normal distribution was not verified, the data were analyzed after log transformation. A small positive number was added prior to the log transformation if the values were negative^71^.

## Data Availability

All data analyzed or generated during this trial are included in this article.
